# A Cluster of Legionnaires' Disease and Associated Pontiac Fever Morbidity in Office Workers, Dublin, June-July 2008

**DOI:** 10.1155/2010/463926

**Published:** 2010-04-15

**Authors:** M. Ward, M. Boland, N. Nicolay, H. Murphy, J. McElhiney, C. Collins, M. Lynch, M. McCarthy, J. O' Donnell

**Affiliations:** ^1^Department of Public Health, Health Services Executive, Dublin, Ireland; ^2^Health Protection Surveillance Centre (HPSC), Dublin, Ireland; ^3^European Programme for Intervention Epidemiology Training (EPIET), Dublin, Ireland; ^4^Mater Misericordiae University Hospital, Dublin, Ireland; ^5^Environmental Health Services, Health Services Executive, Dublin, Ireland

## Abstract

In June and July 2008, two office workers were admitted to a Dublin hospital with Legionnaires' disease. Investigations showed that cooling towers in the basement car park were the most likely source of infection. However, positive results from cooling tower samples by polymerase chain reaction (PCR) did not correlate with subsequent culture results. Also, many employees reported Pontiac fever-like morbidity following notification of the second case of Legionnaires' disease. In total, 54 employees attended their general practitioner or emergency department with symptoms of Legionnaires' disease or Pontiac fever. However, all laboratory tests for Legionnaires' disease or Pontiac fever were negative. In this investigation, email was used extensively for active case finding and provision of time information to employees and medical colleagues. We recommend clarification of the role of PCR in the diagnosis of legionellosis and also advocate for a specific laboratory test for the diagnosis of the milder form of legionellosis as in Pontiac fever.

## 1. Introduction

Legionellosis presents as two distinct clinical entities: Legionnaires' disease (LD) comprises pneumonia with severe multisystem disease and Pontiac fever (PF) which is a self-limiting flu-like illness [1]. 

Annually, on average, 6 cases of LD were reported to the Health Protection Surveillance Centre by the Departments of Public Health in Ireland between 1994 and 2007. This reported rate of two per million is low compared with other European countries, such as Spain and France where rates of 24.8 per million and 22.8 per million were reported, respectively, in 2007. In 2007, the overall European rate of reported infection was 11.4 cases per million [2]. In Ireland, the suggested reason for this low reporting rate is a combination of underdiagnosis and underreporting of LD. During the time period 1994–2007, there were no cases of PF notified nationally in Ireland. 

LD is transmitted from the environment by inhalation of an infectious aerosol [1]. In Europe and Ireland, almost all reported cases of LD are sporadic [2, 3] and the source of the *Legionella* for these cases is rarely determined. However, outbreaks may provide a better opportunity to identify common sources of legionellae. Such outbreaks of LD can be caused by communal sources such as hot tubs, spa pool, hospital, domestic or hotel showers, and wet cooling towers [4]. 

Over a ten-day period in June-July 2008, two cases of LD were reported to the Department of Public Health, Health Services Executive East (HSE-E) in Dublin. Both cases worked in a large Dublin-based insurance firm and were based in a newly built office block in the city. The office building consisted of seven floors over a basement car park. Two cooling towers were located in the basement. The staff smoking area was located immediately adjacent to the cooling towers. Air conditioning within the building was confined to the printing/postal room (3rd floor) and the conference/meeting rooms (6th and 7th floor). This paper describes the cases with LD, the outbreak investigation, and subsequent management. A retrospective cohort study of cases with PF will be reported separately [5].

## 2. Methods

### 2.1. Epidemiological Investigation

The first case of LD was a 58-year-old Southern-European male smoker who worked in the postal department on the 3rd floor of the company office, and became unwell in early June. He was admitted on June 25th to a Dublin hospital with a 3-week history of fever, night sweats, chills, diarrhoea, and confusion. His chest X-ray showed right upper and lower lobe consolidation. Liver function tests and renal indices were elevated. Diagnosis of LD was confirmed by *L. pneumophila* serogroup 1 urinary antigen test (UAT) on the day of admission. He was admitted to the Intensive Care Unit for the management of renal failure associated with LD and was treated with clarithromycin. He was too ill to provide a history on admission. He had no history of travel abroad. He was hospitalised for 14 days. This case was notified to the Public Health Department on June 27th. 

On July 7th, a second case of LD (case 2) was reported to the Department of Public Health. Case 2 was a 48-year-old Irish male nonsmoker, who worked on the 5th floor of the company office. He had been ill and off work for 7 days preceding his presentation to the same Dublin hospital as case 1, with a history of fever, headache, cough, diarrhoea, confusion, and paraesthesia of arms and legs. On admission he was diagnosed with pneumonia and LD was confirmed by UAT on the day of admission. He was treated on the ward with clarithromycin and ciprofloxacin and was discharged home after 10 days. Case 2 lived in a different area of Dublin from case 1. Apart from place of employment there were no other common links such as travel or social activities between both cases. As a result of two linked cases of LD an outbreak control team was formed with representatives from Public Health, Infection Control, Environmental Health, Microbiology, the Health Protection Surveillance Centre, and the Health and Safety Authority.

**Figure 1:**
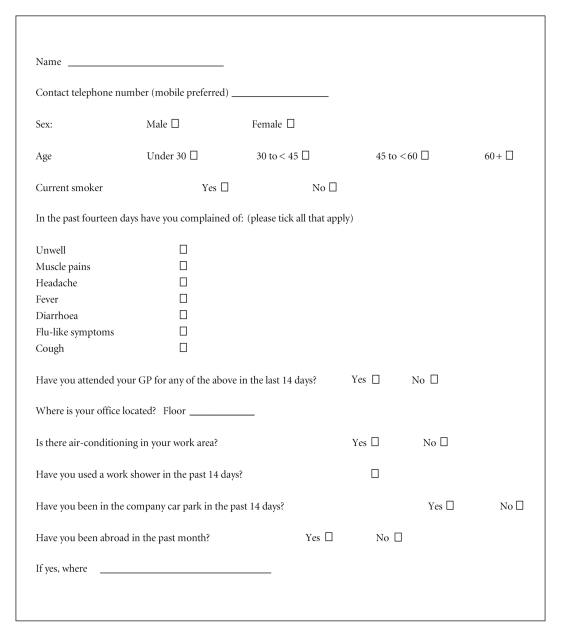
Questionnaire for office workers distributed by email on July 8th 2008.

On July 8th, it was reported to the Department of Public Health HSE-E by company management that many employees were absent from work and had reported severe flu-like illness. Consequently, active case finding was carried out on the morning of July 8th in the company. A standardised questionnaire was emailed to all company and contract employees who had worked in the building at any time during the previous 14 days. Information was requested on symptoms (cough, headache, fever, muscle pains, diarrhoea, and flu-like symptoms), potential exposure (office floor, air-conditioning, and shower use), smoking, travel, and demographics (Figure 1).

### 2.2. Case Definitions

We defined a case of LD as an employee of, or visitor to, the company office who had pneumonia with onset since June 1st, and* Legionella* infection was diagnosed by culture, UAT, or serology (a single high titre or a four-fold rise in specific serum antibody to *L. pneumophila* serogroup 1 or serogroups 2–6 and 8).

We defined a case of PF in the initial investigation as an employee of, or visitor to, the company office with symptoms of fever, headache, myalgia, and nonproductive cough with onset since June 1st. 

### 2.3. Further Investigations

Sickness records were reviewed by Human Resources staff and absent workers were contacted to inform them of the outbreak and to enquire about their health and whether they had sought medical attention. Respondents who met our case definition for LD or PF were promptly telephoned by Department of Public Health medical or nursing staff and advised on appropriate medical follow-up.

Employees who attended the emergency department (ED) or their general practitioner (GP) were followed up to determine their symptomatology, investigation, results, and treatment. Subsequently, a designated clinic was arranged for all employees and contract workers who (a) met our case definition for PF or (b) had attended the ED or their GP with concerns regarding LD. All were invited for *Legionella* serology three weeks after likely exposure. 

In total, 54 employees attended EDs in the Republic of Ireland and Northern Ireland or their general practitioner with symptoms and/or concerns about LD or PF; each had a UAT, all of which were reported negative. Of these at least 38 were reported as having been treated with an antibiotic. Subsequently, 37 of these employees with possible symptoms of PF attended a special clinic for *Legionella *serology. No employee tested showed serological evidence of infection with *L. pneumophila *serogroups 1–6 and 8, three weeks after likely exposure. The serological tests were performed by the legionella reference services at the Respiratory & Systemic Infection Laboratory, HPA Centre for Infections, Colindale, UK.

### 2.4. Environmental Investigation

On July 8th, Public Health doctors met on site with the company's Health and Safety manager, Risk Assessment manager, and Human Resources manager. Public Health doctors, Environmental Health officers of the Health Services Executive, and Environmental Health officers of Dublin City Council conducted an inspection and environmental risk assessment of the building. On July 9th, the Health, and Safety Authority, the organisation responsible for ensuring safety, health and welfare at work, inspected the building. There were two separate water-containing systems supplying the office. The primary system consisted of two cooling towers located in the large basement carpark of the building. Both cooling towers were immediately adjacent to the staff smoking area. This system was used to chill incoming water. The secondary system was a hot and cold water system used to service sinks and showers. There was no physical connection between the primary and secondary systems. Both systems were supplied with mains water. Managers reported the use of an alarmed temperature control system for the water supply to the building. There was no fountain in the building. 

As a precautionary measure, after case 1 was reported and prior to any treatment, multiple water samples were taken from both water systems and sent for PCR and culture for *Legionella *spp. Mains water was checked at points entering the building. Samples from tower 1 were positive by PCR for *Legionella* spp. but below detection level on culture (Table 1). Samples from tower 2 were positive by PCR for *Legionella *spp. at lower levels than tower 1. However, *L. pneumophila* was cultured from tower 2 at 100 colony forming units per litre (CFU/L). Levels of* L. pneumophila *detected by PCR from the showers/sinks tested were below the threshold level for clinical significance defined by the chemical company, and *L. pneumophila* was not cultured from any showers/sinks tested.

Both cooling towers were immediately deactivated, cleaned, and disinfected as recommended [6]. The cooling towers have remained inactive and are now replaced by a dry cooling system. 

### 2.5. Microbiological Investigations

Both patients admitted to hospital were confirmed cases of LD by *L. pneumophila *serogroup 1 UAT (Binax). Sputum samples from both patients, after they had commenced antibiotics, did not culture *Legionella *species. However sputum from case 2 sent to the legionella reference services at the Respiratory Systemic Infection Laboratory HPA was positive by PCR testing for *L. pneumophila* DNA. However, there was insufficient DNA to allow sequence typing of the strain. The environmental isolate from cooling tower 2 was identified as *L. pneumophila* serogroup 1, monoclonal antibody subgroup “Knoxville”.

### 2.6. Communication Methods

After the first case of LD was notified, an email alert was sent to 600 company employees informing them of the symptoms of LD and advising them to seek medical attention from their general practitioner if they had any concerns regarding illness. This alert was repeated to all employees three days later. In addition, every line manager endeavoured to contact their employees to confirm they had received relevant public health information of LD and were not symptomatic. Similar efforts were also made to check on contract employees and visitors to the company since June 1st. Following reporting of the second case of LD, an alert was sent to all general practitioners and ED consultants in the region by fax and email. In addition, due to the high level of concern among the employees in the company, two educational/information sessions on LD were held in the office by medical doctors.

## 3. Discussion

This paper describes a cluster of LD among office workers in Dublin. The only common link among the two cases was their place of employment. Consequently, the most likely source of this cluster was the cooling towers in the basement car park. Definitive microbiological confirmation of the cooling towers as the source of infection required an isolate from one of the patients to match the environmental isolate. As *L. pneumophila* cultured from cooling tower 2 was typed as serogroup 1 mAb2+ve which is a recognised cause of clinical infection and also a serogroup detected by UAT, we can reasonably conclude that cooling tower 2 was the most likely source of this cluster. At present, Ireland does not have legislation requiring the registration of cooling systems at national level [6]. A system of statutory notification by the owner/occupier of such high risk sites, as recommended in recently published National Guidelines, would facilitate investigation and control of future cases/outbreaks of legionellosis [2, 6]. 

Early identification and treatment of patients with LD have important implications for clinical management of patients and outcome [7]. Studies comparing the clinical manifestations of *Legionella* pneumonia to other types of pneumonia indicate that LD is not “atypical” and clinical features such as diarrhoea and confusion are not sufficiently distinctive to distinguish LD from other causes of community-acquired pneumonia. Consequently, in order to increase awareness of LD as a possible diagnosis, after the initial case of LD was confirmed we immediately corresponded with company employees, management, as well as GP and hospital colleagues. Several outbreaks have observed a relationship with the dose of inoculum. For example, an outbreak has been described where two workers in a cooling tower with different levels of exposure developed pneumonia and PF, respectively [3]. Immunological conditions and underlying disease are among the factors that affect susceptibility to legionellosis [8, 9]. Such factors may explain the broad spectrum of clinical presentation in our investigation where the majority of employees reported no symptoms, a substantial number reported a mild flu-like illness, and two employees developed LD.

Few studies have addressed the practical value of PCR in routine clinical and environmental microbiology laboratories. PCR offers theoretical advantages, such as a high sensitivity, rapid availability of results, and the potential to detect infections caused by various serogroups of *L. pneumophila* as well as by nonpneumophila species [1, 10, 11]. Conversely, the usefulness of PCR is limited by its inability to distinguish between live, viable nonculturable and dead *Legionella* cells. In this cluster, while PCR provided time information on the number of *Legionella *in the environmental samples tested, equivalence with the number of CFU by culture was not found. Our results were similar to previous studies which showed that PCR results are usually higher than culture values [11, 12]. It could be argued that PCR is too sensitive a method that detects viable nonculturable and dead *Legionella *cells that may not necessarily be pathogenic and thus PCR is not an accurate measure of a real health risk. Nevertheless, in this outbreak, detection of *Legionella* species by PCR was a marker of environmental contamination by *Legionella* and led to preventive measures. As culture of *Legionella* remains the “gold-standard” in diagnosis of LD [2], we suggest that PCR should complement rather than replace traditional culture methods in routine clinical practice and in monitoring industrial cooling water systems until its use is further validated. 

The affected premises in this cluster ware a new purpose built “green building” which was occupied for three months prior to the reported onset of illness. A green building is defined as “the practice of increasing the efficiency with which buildings use resources—energy, water, and material—while reducing the building's impact on human health and the environment during the building's lifecycle, through better siting, design construction, operation, maintenance, and removal” [13]. However, despite this aim, it is likely that malfunctioning of the cooling towers' microbiological control programme was the source of employees' illness in this outbreak. Illness because of exposure to some unseen agent in a workplace can cause alarm out of proportion to the risk [14]. In this outbreak, the relative importance of panic and illness among workers were difficult to determine. However, the reported severity, similarity, and duration of symptoms among staff supported the diagnosis of a PF-like illness. This diagnosis was based on an epidemiological link and similar symptomatology in workers. Absence of specific laboratory diagnostics for PF mitigated against differentiation of psychological and pathological causes of reported staff illness. Improvements in diagnostic testing could lead to a better understanding of outbreaks of PF [15] and also help differentiate this illness from other causes of influenza-like illness in order to direct consequent appropriate remedial action.

The nonspecific presentation of LD makes clinical diagnosis very difficult in most patients with community-acquired pneumonia of uncertain aetiology. Thus, the key to diagnosis is appropriate microbiological testing [1]. Both patients in this cluster were diagnosed by UAT. This test detects *L. pneumophila *serogroup 1 antigens in a urine sample within one hour with a specificity of 95% [9]. In Ireland, in 2007, UAT was the principal diagnostic method used for 75% of LD cases [2]. Sensitivity of UAT is associated with the clinical severity of disease [1]. Thus, patients with milder disease like PF may have been underdiagnosed if UATs alone were used. Such a scenario has been previously suggested by authors who claim that serology in cases of PF is not consistently positive and also that the presence of urinary antigen is not systematic in detecting *L. pneumophila* serogoup 1 among PF patients [16–19]. Indeed many prior studies of UATs in PF have very low yields [19]. This may be because these laboratory tests were primarily developed and used for diagnosis, of LD in hospitalised patients. Thus, the validity of these tests for those with mild clinical illness, not requiring hospital admission, has not been established. 

This investigation demonstrated that timely intervention is vital in control of a cluster of legionellosis. In order to control, and ideally prevent, future clusters of legionellosis we recommend the statutory notification of high risk sites and also improved diagnostic tests.

## Figures and Tables

**Table 1:** Microbiological results from cooling towers 1 and 2 by PCR and culture, Dublin, July 2008.

Cooling Tower	PCR (GU/L)		Culture (CFU/L)		
	*Legionella* spp.	*L. pneumophila*	*Legionella* spp.	*L. pneumophila* serogroup 1	*L. pneumophila* serogroup 2–14
1	61,800,000	73,200,000	< Detection level	< Detection level	< Detection level
2	174,000	<3,300	100	100	< Detection level
